# The Effect of Tuberculosis on Mortality in HIV Positive People: A Meta-Analysis

**DOI:** 10.1371/journal.pone.0015241

**Published:** 2010-12-30

**Authors:** Masja Straetemans, Ana L. Bierrenbach, Nico Nagelkerke, Philippe Glaziou, Marieke J. van der Werf

**Affiliations:** 1 Unit Research, KNCV Tuberculosis Foundation, The Hague, The Netherlands; 2 Department of Clinical Epidemiology, Biostatistics and Bioinformatics, Center for Infection and Immunity Amsterdam (CINIMA), Academic Medical Center, Amsterdam, The Netherlands; 3 Stop TB Department, World Health Organization, Geneva, Switzerland; 4 Department of Community Medicine, United Arab Emirates University, Al Ain, United Arab Emirates; McGill University, Canada

## Abstract

**Background:**

Tuberculosis is a leading cause of death in people living with HIV (PLWH). We conducted a meta analysis to assess the effect of tuberculosis on mortality in people living with HIV.

**Methods:**

Meta-analysis of cohort studies assessing the effect of tuberculosis on mortality in PLWH. To identify eligible studies we systematically searched electronic databases (until December 2008), performed manual searches of citations from relevant articles, and reviewed conference proceedings. Multivariate hazard ratios (HR) of mortality in PLWH with and without tuberculosis, estimated in individual cohort studies, were pooled using random effect weighting according to “Der Simonian Laird method” if the p-value of the heterogeneity test was <0.05.

**Results:**

Fifteen cohort studies were systematically retrieved. Pooled overall analysis of these 15 studies estimating the effect of tuberculosis on mortality in PLWH showed a Hazard Ratio (HR) of 1.8 (95% confidence interval (CI): 1.4–2.3). Subanalysis of 8 studies in which the cohort was not exposed to highly active antiretroviral therapy (HAART) showed an HR of 2.6 (95% CI: 1.8–3.6). Subanalysis of 6 studies showed that tuberculosis did not show an effect on mortality in PLWH exposed to HAART: HR 1.1 (95% CI: 0.9–1.3).

**Conclusion:**

These results provide an indication of the magnitude of benefit to an individual that could have been expected if tuberculosis had been prevented. It emphasizes the need for additional studies assessing the effect of preventing tuberculosis or early diagnosis and treatment of tuberculosis in PLWH on reducing mortality. Furthermore, the results of the subgroup analyses in cohorts largely exposed to HAART provide additional support to WHO's revised guidelines, which include promoting the initiation of HAART for PLWH co-infected with tuberculosis. The causal effect of tuberculosis on mortality in PLWH exposed to HAART needs to be further evaluated once the results of more cohort studies become available.

## Introduction

People living with HIV (PLWH) are estimated to have a 20 times higher risk on developing tuberculosis (TB) disease compared to people living without human immunodeficiency virus (HIV) infection in countries with an HIV prevalence of at least 1%.[1] Fifteen percent of the incident TB cases in 2008 are estimated to be co-infected with human immunodeficiency virus (HIV).[1] TB is the leading direct cause of death among PLWH in Africa and a major cause of death elsewhere.[2], [3] The estimated number of incident TB cases in PLWH was 1.4 million in 2008 (range 1.3–1.5 million). An estimated 0.52 million (range 0.45–0.62 million) TB deaths occurred in 2008 among PLWH (38%, range 31%–45%).[1] Africa accounted for 79% of the HIV-positive TB cases, followed by South-East Asia with 13%.[1]

In PLWH, HIV infection increases the risk of progressing from TB infection to TB disease.[4] Furthermore, TB may act as cofactor in the progression of HIV infection by increasing the HIV viral load through inducing a faster HIV replication and/or by contributing to a reduction in the CD4 cell count.[5] The widespread use, since 1996, of highly active antiretroviral therapy (HAART) has substantially improved the prognosis of HIV-infected patients both in industrialised and low-income settings [6] and survival in HIV/TB co-infected individuals.[7], [8]

Although there seems to be consensus that TB does accelerate HIV replication, the impact of TB disease on HIV disease progression at the population level is less clear.[9] According to the International Classification of Diseases (ICD-10) deaths from TB in PLWH are classified as HIV deaths.[3] [10] Previously, a non systematic pooled analysis showed that TB was weakly associated with an increased risk of death in PWLH (relative risk: 1.1; 95% confidence interval (CI): 1.0–1.2).[11] This non-systematic pooled analysis assessed the effect of various exposures (TB or TB as an AIDS defining condition) on various endpoints (mortality in PLWH or mortality in PLWH having developed AIDS during follow up period), which may have substantially increased heterogeneity. The aim of our meta-analysis was to assess the effect of TB on mortality in a broad cross-section of the population of PLWH.[12]

## Methods

A protocol was developed in advance of conducting this systematic review and meta-analysis.

### Search strategy and selection criteria

To identify cohort studies assessing the effect of TB on mortality in PLWH we searched for publications in the PubMed, Embase and Scopus databases through December 2008. The combination of key words (exploded MESH headings and free text terms) in the search strategy included HIV Infections, AIDS-Related Opportunistic Infections, Acquired Immunodeficiency Syndrome, cohort study, tuberculosis, mortality, survival, HIV, AIDS. Furthermore, reference listing of eligible studies was conducted and we hand-searched abstracts of relevant TB and AIDS conferences till 2008. We contacted authors of eligible studies to identify additional published and unpublished studies. Identified studies were reviewed for eligibility by two authors (MS, MvdW) based on title and abstract. Eligible studies were cohort studies assessing the effect of TB on mortality in a HIV positive cohort by calculating multivariate hazard ratios (HR) and corresponding 95% CI through Cox Proportional Hazard models. Studies not fulfilling the eligibility criteria, studies defining the cohort on AIDS status, and studies not reporting a mortality rate (e.g. only included death PLWH) were excluded. A priori we did not exclude non-English articles but all full text articles of the potential eligible studies appeared to be in English.

### Data extraction

One reviewer (MS) extracted data from all eligible studies and a second reviewer (MvdW) independently extracted data from a subset of articles. Data extraction included information on study setting, study population, cohort size, duration of follow up, antiretroviral therapy (ART), baseline CD4 cell count, baseline HIV viral load, exclusion of patients with TB history, mortality, type of statistical model and measures of association estimating the effect of TB on mortality. Authors of six included studies were approached to provide additional information or asked for clarification if needed.

### Quality assessment

Quality assessment of eligible studies was done according to the Newcastle-Ottawa Quality Assessment Scale for cohort studies.[13] This scale seemed most appropriate for our purpose and does include important quality features of cohort studies.[14], [15] We did not assess the study quality of two studies [16], [17] because the full text was unavailable at time of submission of this manuscript.

### Quantitative data synthesis

Log HR of the multivariate analyses in the individual cohort studies were combined using the inverse variance method. Heterogeneity across studies was estimated by calculating I^2^. Random effect weighting according to the Der Simonian Laird method [18] was conducted when the p-value of the heterogeneity test was <0.05. Four studies estimated the multivariate HR by marginal structural Cox proportional hazard models and standard Cox proportional hazard models.[8], [11], [19], [20] For the main analyses we pooled the results obtained by the standard Cox proportional hazard model to be more consistent with the other studies that only estimated the multivariate HR by standard Cox proportional hazard model. The stratified analyses of two studies not reporting an overall estimate were included as separate studies.[21], [22] Because these stratified analyses include different individuals we are not subject to double counting of data.[23] To prevent too much weight of one study assessing mortality separately in HIV1 and HIV2 positives [21] we only included the stratified analyses assessing effect of TB on mortality in HIV1 positives.

The eligible studies included TB patients who either had TB diagnosis confirmed at study entry (‘prevalent TB patients’); patients who did not have TB at study entry but who developed TB during the course of the study (‘incident TB patients’) and studies including both ‘prevalent’ and ‘incident’ TB patients. ‘Incident TB’ did not necessarily indicate a new episode of TB in a person without prior TB, it might also indicate a recurrence of TB as not all studies have excluded PLWH with a prior history of TB. In the main analyses we have estimated the effect of ‘incident’, ‘prevalent’ and ‘incident’+‘prevalent’ TB on mortality. In the secondary analyses we estimated pooled HRs in the following subpopulations:

- Baseline CD4 cell count <200 µ/L;
- Studies conducted before the introduction of HAART (<1996) or reporting that less than 10% of the HIV positives had initiated HAART;
- Studies conducted in 1996 or thereafter and reporting ≥10% or ≥50% HAART initiation by PLWH;
- Studies conducted in the USA, Africa, Asia and Europe.

Sensitivity analyses were conducted excluding studies with lower quality according to the quality assessment tool. Publication bias was assessed by a scatter plot (funnelplot) of the log HR (x-axis) versus precision defined as 1/standard error (y axis) of the 15 studies included in the main analyses. Analyses were conducted with STATA 10.

## Results

### Selection of included studies

**Figure 1:**
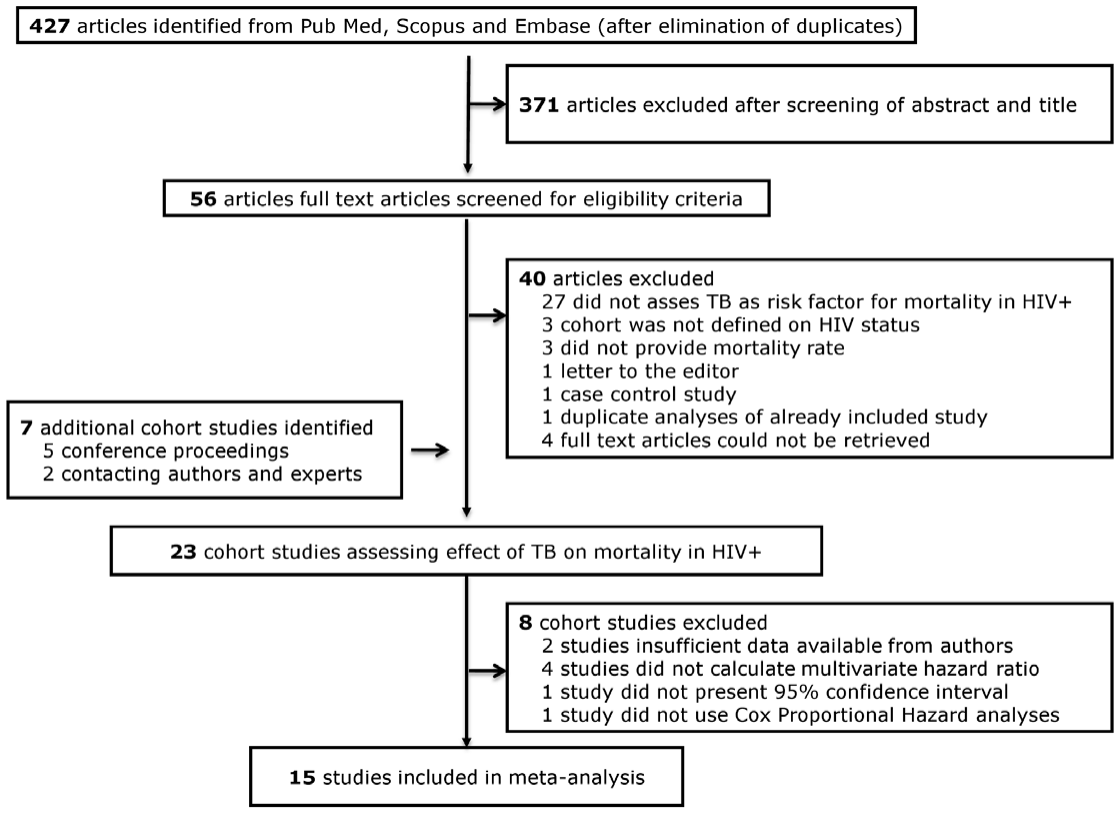
Flow diagram of papers accepted and rejected during selection procedure.

**Figure 2:**
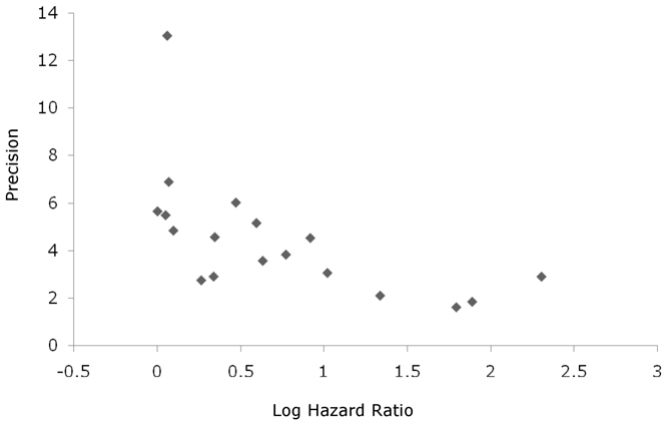
Funnelplot of 15 studies estimating the effect of tuberculosis on either all cause mortality or AIDS-related mortality in people living with HIV. Points indicate log hazard ratios (x-axis) from 15 studies (contributing to 18 separate hazard ratio's) assessing the effect of “prevalent”, “incident”, and “prevalent+incident” TB on AIDS-related/all cause mortality in HIV positive individuals. Precision is calculated as 1/standard error. A lower precision indicates a more accurate estimate.

We identified 15 cohort studies comparing mortality in PLWH with and without TB that were eligible for our meta-analysis (Figure 1). The funnel-plot (Figure 2) suggests a lack of publications on small sized studies that did not show an effect of TB on mortality in PLWH.

### Study characteristics

Table S1 presents the study characteristics of the individual studies. Ten [8], [11], [19]–[22], [24]–[27] of the 15 included cohort studies that were prospective and five were retrospective [16], [17], [28]–[30]. The majority of the studies (87%) included both HIV positive males and females recruited from hospitals, primary care units and research institutes[8], [16], [17], [19], [21], [22], [24]–[29], [31] while 2 studies included either only males[11] or females [20] recruited from similar institutions.

Information on TB diagnosis could be obtained for 11 studies. In 9 studies TB was defined as sputum smear positive confirmed by culture (definite TB) or clinical and radiologic improvement after TB treatment (probable TB), in a patient with clinical signs and symptoms consistent with active TB. [11], [20], [22], [28], [21], [26], [27], [29], [31] In 2 studies TB was primarily diagnosed by sputum smear examination and clinical symptoms and/or radiological abnormalities without culture confirmation. [8], [16] PLWH with prior TB diagnosis were excluded from participation in five studies [11], [20], [22], [28], [29] while in nine studies TB history was not an exclusion criteria [8], [16], [17], [19], [24], [25], [27], [31], [33] and one study did not report on TB history.[26] Ten studies were conducted during or after 1996, the era coinciding with availability of HAART [8], [11], [16], [17], [19]–[21], [24]–[26] of which eight reported that part of the cohort(s) had initiated HAART.[8], [11], [16], [17], [20], [24], [25], [32] In four studies all PLWH initiated HAART.[19], [24]–[26]

Table S2 presents the individual results of studies assessing the effect of TB on mortality in PLWH. Ten studies [11], [16], [20]–[22], [26]–[29], [31] reported in at least one of their analyses a statistically significantly increased hazard on mortality of ≥1.5 in PLWH with TB compared to PLWH without TB (Table S2).

### Meta-analysis

#### Main results

**Figure 3:**
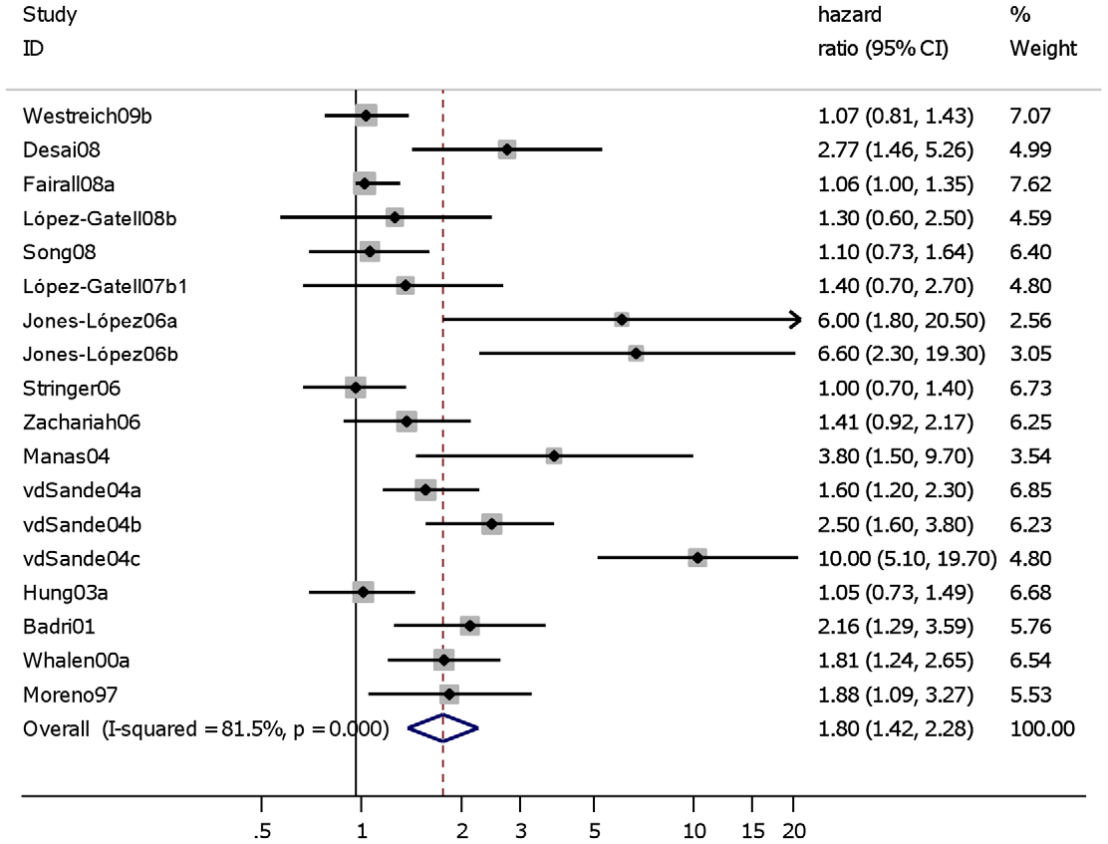
Studies assessing the effect of “prevalent”, “incident” and “prevalent”+“incident” TB on AIDS-related/all cause mortality in HIV positive individuals. Study ID on the Y-axis refers to first author and publication year; % weight refers to influence of each study on overall estimate (weights are from random effect analyses); for each study the central diamond indicates multivariate hazard ratio, line represents 95% confidence interval (CI), and the grey square reflects the study's weight in the pooling; overall estimate refers to pooled estimate of hazard ratio after mathematical combination of all studies; the X-axis indicates the scale and the direction of the effect of tuberculosis on mortality in HIV positive individuals. I-squared denotes the extent of heterogeneity in study outcomes, with a (hypothetical) value of 100% meaning considerable heterogeneity and 0% meaning no heterogeneity between studies.

Including all 15 studies that estimated the effect of TB on either all cause mortality or AIDS-related mortality in PLWH showed a pooled HR of 1.8 (95% CI: 1.4–2.3) (Figure 3). Excluding one study that reported on AIDS-related mortality instead of all cause mortality [11], the estimated effect of TB on mortality in PLWH was similar (HR: 1.8; 95% CI: 1.4–2.3) (Table 1, Analyses 2.1). Excluding three studies in which TB was only measured at study entry (‘prevalent TB’) [16], [19], [25] showed similar results (HR: 1.9 (95% CI: 1.5–2.6) (Table 1, Analyses 2.2). Limiting the analyses to those five HIV positive cohorts without TB at study entry but estimating the effect of ‘incident’ TB on all cause mortality in HIV positives, risk of mortality was 2.6 times higher for HIV positives who developed TB compared to HIV positives who did not develop (HR: 2.6 (95% CI: 1.6–4.1)). (Table 1, Analyses 3.1).

**Table 1:** Pooled multivariate hazard ratios among subgroups of studies estimating the effect of TB on all cause mortality in HIV positive individuals.

					Heterogeneity		
	Analyses	N Studies	N separate HRs	Pooled Hazard Ratio (95% CI)	p *	I^2^	References
**Timing of TB diagnosis**							
“prevalent”, “incident”, “prevalent+incident”	2.1	14	17	1.8 (1.4–2.3)	0.00	82.6%	[8], [16], [17], [19], [20], [21], [22], [24], [25], [26], [27], [28], [29], [31]
“incident”, “prevalent+incident”	2.2	11	14	1.9 (1.5–2.6)	0.00	84.6%	[8], [17], [20], [21], [22], [24], [26], [27], [28], [29], [31]
“incident”	3.1	5	8	2.6 (1.6–4.1)	0.00	87.7%	[8], [20], [21], [22], [29]
“prevalent”	3.2	3	3	1.5 (0.9–2.2)	0.03	72.6%	[16], [19], [25]
“prevalent + incident”	3.3	5	5	1.1 (1.0–1.2)	0.12	45%	[8], [17], [24], [26], [27]
**CD4 ≤200 cells/µL**							
	4	3	6	1.5 (1.1–2.1)	0.01	70.4%	[19], [21], [26]
**Year + % report HAART**							
<1996 or report NO HAART	5.1	8	11	2.6 (1.8–3.6)	0.00	73%	[20], [21], [22], [26], [27], [28], [29], [31]
<1996 or ≤10% report HAART	5.2	9	12	2.6 (1.9–3.5)	0.00	70.7%	[16], [20], [21], [22], [26], [27], [28], [29], [31]
≥1996+≥10%	5.3	7	7	1.1 (0.99–1.3)	0.90	0.0%	[8], [17], [19], [20], [24], [25], [26]
≥1996+≥50%	5.4	6	6	1.1 (0.9–1.3)	0.75	0.0%	[17], [19], [20], [24], [25], [26]
**Geographic region**							
USA	6.1	2	2	2.4 (1.3–4.3) †	0.95	0.0%	[11], [20]
Africa	6.2	8	11	2.2 (1.6–3.2) ‡	0.00	84.1%	[16], [19], [21], [22], [24], [25], [27], [31]
Asia	6.3	2	2	1.1 (0.8–1.4)	0.87	0.0%	[17], [26]
Europe	6.4	3	3	1.8 (0.95–3.3)	0.01	76.9%	[8], [28], [29]
**Sensitivity analyses** §							
Excluding scoring 0 or 1 on ‘comparability’	S1	9	11	1.6 (1.2–2.1)	0.00	84.6%	[8], [19], [20], [21], [24], [25], [26], [27], [31]
Excluding scoring 0 or 1 on ‘outcome’	S2	9	12	1.9 (1.4–2.6)	0.00	82.9%	[19], [20], [21], [22], [24], [25], [26], [29], [31]
Excluding overall score <67%	S3	10	13	1.8 (1.3–2.4)	0.00	84.6%	[8], [19], [20], [21], [22], [24], [25], [26], [29], [31]

*The weights are from the random effect analyses if p<0.05 and the weights are from the fixed analyses if p≥0.05;

† The HR indicates the effect of TB on all cause/AIDS related mortality because López Gattell, et al (2008) ^11^ reports the effect of TB on AIDS-related mortality;

‡ For both studies the multivariate hazard ratios as assessed by the marginal structural Cox proportional hazard have been included;

§ Analyses number 2.1 formed the basis for the sensitivity analyses.

### Results of secondary and sensitivity analyses

#### CD4 cell count

**Figure 4:**
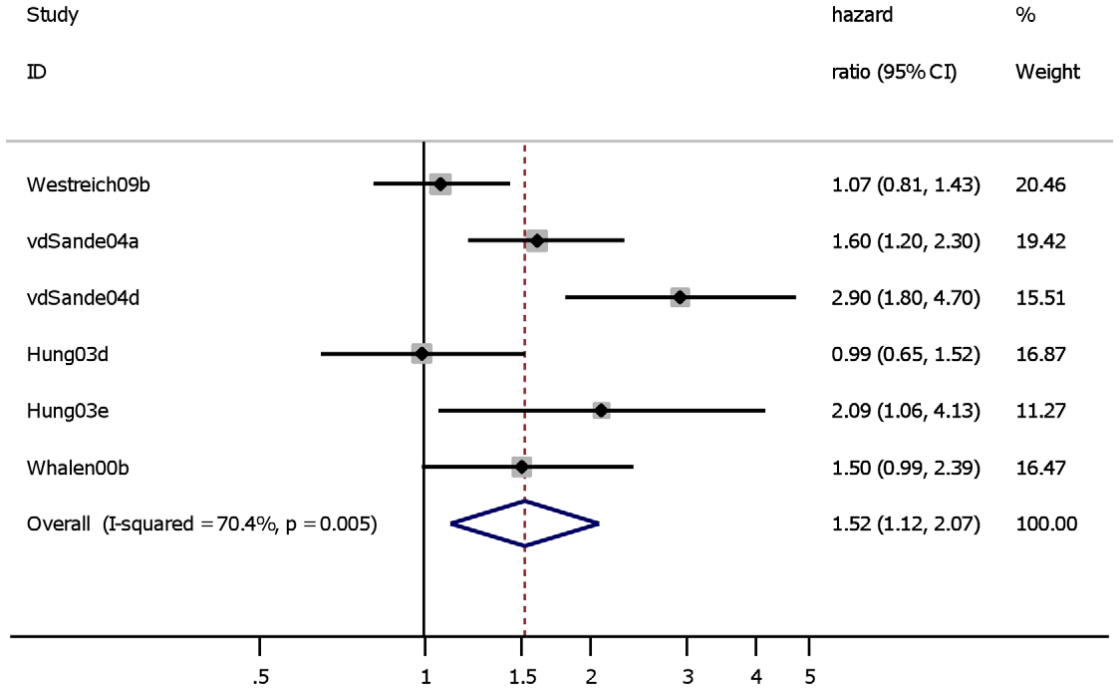
Studies assessing the effect of “prevalent”, “incident” and “prevalent”+“incident” TB on all cause mortality in HIV positive individuals with baseline CD4 cell count ≤200 cells/µL. Study ID on the Y-axis refers to first author and publication year; % weight refers to influence of each study on overall estimate (weights are from random effect analyses); for each study the central diamond indicates multivariate hazard ratio, line represents 95% confidence interval (CI), and the grey square reflects the study's weight in the pooling; overall estimate refers to pooled estimate of hazard ratio after mathematical combination of all studies; the X-axis indicates the scale and the direction of the effect of tuberculosis on mortality in HIV positive individuals.

Four studies either included only PLWH with baseline CD4 cell count ≤200 cells/µL [19] or conducted stratified analyses [21], [26], [31] to assess the effect of TB (‘prevalent’, ‘incident’, ‘prevalent+incident’) on mortality in PLWH with baseline CD4 cell count ≤200 cells/µL. Across these four studies, the pooled effect of TB on mortality among PLWH with CD4 ≤200 cells/µL (HR 1.5; 95% CI: 1.1–2.1) was similar to that among PLWH including all CD4 levels (Figure 4).

#### HAART

**Figure 5:**
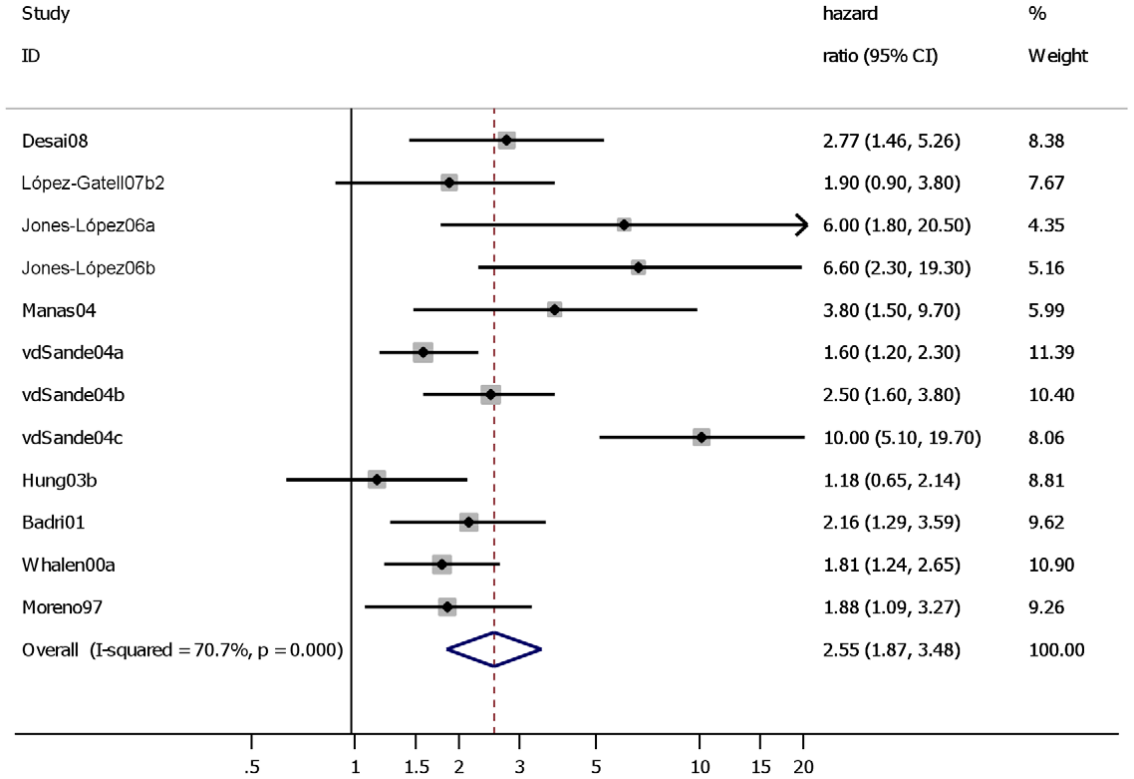
Studies assessing the effect of “prevalent”, “incident” and “prevalent”+“incident” TB on all cause mortality in HIV positive individuals before HAART era (<1996) or ≤10% of cohort has reported use of HAART. Study ID on the Y-axis refers to first author and publication year; % weight refers to influence of each study on overall estimate (weights are from fixed effect analyses); for each study the central diamond indicates multivariate hazard ratio, line represents 95% confidence interval (CI), and the grey square reflects the study's weight in the pooling; overall estimate refers to pooled estimate of hazard ratio after mathematical combination of all studies; the X-axis indicates the scale and the direction of the effect of tuberculosis on mortality in HIV positive individuals.

**Figure 6:**
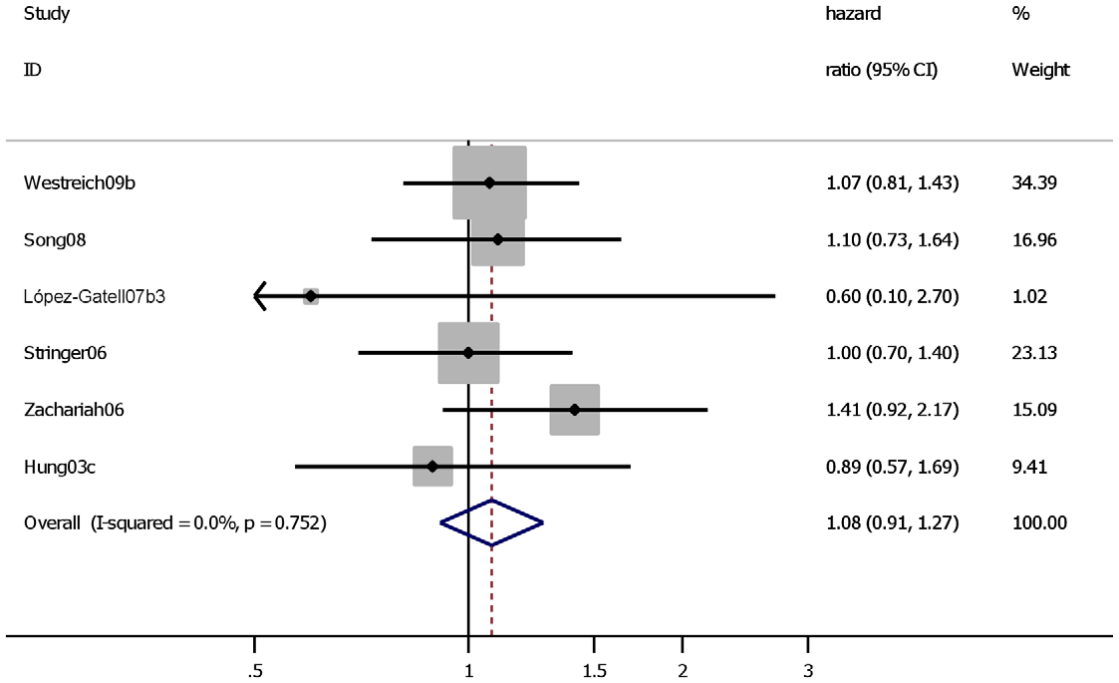
Studies assessing the effect of “prevalent”, “incident” and “prevalent”+“incident” TB on all cause mortality in HIV positive individuals during HAART era and ≥50% of cohort has reported use of HAART. Study ID on the Y-axis refers to first author and publication year; % weight refers to influence of each study on overall estimate (weights are from fixed effect analyses); for each study the central diamond indicates multivariate hazard ratio, line represents 95% confidence interval (CI), and the grey square reflects the study's weight in the pooling; overall estimate refers to pooled estimate of hazard ratio after mathematical combination of all studies; the X-axis indicates the scale and the direction of the effect of tuberculosis on mortality in HIV positive individuals.

TB had a large effect on mortality in PLWH participating in a study conducted before HAART was available: HR 2.6 (95% CI: 1.8–3.6). (Table 1, Analyses 5.1) When including one study that was conducted during the HAART era but of which less than 10% of the cohort had been exposed to HAART resulted in a similar HR: 2.6 (95% CI: 1.9 to 3.5) (Figure 5). In contrast, the subset of studies in which at least 50% of the cohort was reported to be exposed to HAART did not show an effect of TB on mortality: HR 1.1 (95% CI: 0.9–1.3) (Figure 6).

#### Geographical region

Stratifying the studies per region showed that TB was significantly associated with an increased hazard of mortality in PLWH in the USA and Africa and reaching statistically significance in Europe (Table 1, Analyses 6.1–6.4). For Asia, no increased TB-associated mortality risk was observed based on the results of only two studies. [17], [26]

The three sensitivity analyses showed that excluding studies with relatively low scores on the quality assessment scale resulted in similar HRs (Table 1, Analyses S1–S3).

## Discussion

This meta-analysis shows that PLWH with TB face an approximately two times higher risk of death from all causes compared to PLWH without TB. The increased hazard of mortality implies that PLWH with TB die earlier compared to PLWH without TB.

Diagnosing TB in PLWH is difficult due to the more frequent presentation with atypical symptoms and the increased proportion of both smear-negative pulmonary TB and extra pulmonary TB.[4], [34]–[37] Apart from these difficulties, access to health services and treatment in high HIV prevalence resource constrained settings is restricted due to an increased demand on over-stretched and under-resourced TB services [38], which may also be geographically out of reach for a significant proportion of the affected population.[34] These factors may result in late diagnosis, more advanced TB, delay and/or lack of specific TB treatment and poorer TB treatment outcomes contributing to a pronounced mortality effect of TB in PLWH. [4], [34], [35], [39]

The increased risk of dying in PLWH with TB disease may, instead of dying from TB, be due to HIV-related conditions.[38], [40] The development of incident TB is indicative of a poor immune recovery. The poor prognosis following a diagnosis of TB suggests that TB in PLWH could be considered as a proxy for severe immunosuppression, with associated poor survival [40] which may be the consequence of an increased risk of dying from HIV-related conditions.[38] In five studies assessing cause of mortality and including PLWH initiating TB treatment, the percentage of HIV positive TB patients dying during TB treatment from TB varied from 16% to 46% of the total HIV positive deaths during TB treatment.[41]–[45] These results indicate that more than half of the HIV positive TB patients, TB is not the main cause of death. Other main causes of death included pneumonia [41], [42], [45], gastro intestinal disease [44], [45], wasting syndrome, Kaposi's sarcoma [41], [44], meningitis [44], (other) opportunistic infections [41], [42] [44], toxic epidermal necrolysis [45], miscellaneous [41] or unknown causes.[41], [42], [44], [45]

Our meta-analysis results indicate that in cohorts of PLWH of which at least 50% of the cohort had been exposed to HAART, TB did not seem to have a higher risk of death compared to those PLWH without TB (HR 1.1; 95% CI: 0.9–1.3). Several reasons may explain why we did not find an effect of TB on mortality in the subset of patient populations with high HAART use. We further discuss the post hoc conclusion of our subgroup analyses.[46] The first possibility is that the ‘true’ effect of TB on mortality in PLWH in advanced stages of HIV infection may have been obscured, because of their high risk of dying from other HIV-related conditions.[42] It is also possible that PLWH who survived early death related to TB were more likely, compared to TB patients who died before initiating HAART, to benefit from HAART due to increased CD4 cell count after HAART initiation.[8], [47] This may possibly result in reductions of risks for major life threatening opportunistic infections [48] that has been associated with lower mortality hazard.[49] For the majority of the included studies CD4 cell count was only measured at baseline therefore the hypothesized clinical treatment success of HAART, as expressed in increase of CD4 cell count, could not be determined based on our results. PLWH initiating HAART may have been taking anti-TB therapy for weeks which might have reduced the survival difference between PLWH with and without TB.[24] Timing and duration of TB treatment could not be determined for most of the studies [17], [19], [24]–[26] in which ≥50% of the PLWH initiated HAART. Previously, studies have reported a reduction in TB related mortality in PLWH initiating HAART.[50], [51] These results are in line with the results of our meta-analysis. A retrospective cohort study on PLWH and TB of which one group started with HAART after initiating TB treatment reported that most observed deaths were due to non TB related conditions.[50] Another cohort study showed that among PLWH, TB-related mortality tended to be lower in PLWH receiving HAART compared to PLWH without HAART.[52] Our findings that PLWH and TB did not seem to have an higher risk on mortality compared to those PLWHI without TB in cohorts largely exposed to HAART can also be explained by selection bias, if individuals most prone to dying from TB have already died before initiating HAART. This could have resulted in an underestimation of mortality associated with TB. However, we do not think that such selection bias had a large impact on our pooled estimate as in three studies PLWH still developed TB after initiating HAART.[17], [24], [26] Information bias may have masked the effect of TB on mortality in PLWH during the HAART era due to under diagnosis or misdiagnosis of TB patients. Support for this hypothesis is the fact that two studies reported that TB diagnosis was only conducted at HAART initiation and compared mortality in PLWH and TB at HAART initiation to mortality in PLWH without TB at HAART initiation.[19], [25] Individuals in this latter group may have developed TB but this has not been diagnosed because of the specific study design. Furthermore, one study [24] specifically mentions that new TB infections occurring while patients are receiving ART had not been well diagnosed in the specific setting. In addition, misclassification of TB patients in this specific HAART setting may have occurred since culture and smear results from the primary healthcare clinics were lacking in one study [19] and TB diagnosis is more difficult in the last stage of immunodeficiency in patients who are severely malnourished and may present with atypical symptoms.[25] A final hypothetical explanation for an absence of TB excess mortality in HAART cohorts is that TB may be the reason for diagnosing HIV and consequently initiating HAART. Thus in the HAART era PLWH may benefit from their TB because it could be a reason for starting HAART earlier thereby stopping and or slowing their decline to death.

### Possible biases related to meta-analysis

The interpretation of the funnel plot suggested a lack of smaller sized studies not showing an effect of TB on mortality in PLWH. This may imply that the results of this meta-analysis are overestimated. However, other sources of biases may have counterbalanced this effect by underestimating the effect of TB on mortality. By using Cox Proportional Hazard analyses the individual HRs may well have been biased because of lack of adjustment for time dependent confounders in the analyses.[53] When assessing the impact of TB on mortality in PLWH, CD4 cell count may act as time dependent confounder. Lower CD4 cell counts are associated with increased risk of mortality [4], [31], [47], [49], [54]–[56] but also with increasing incidence of TB [57]–[60] while the presence of TB disease is associated with reduced CD4 cell counts.[19] Consequently, a lower CD4 cell count not only predicts and increases the risk of mortality but also influences the likelihood of developing TB and can play an intermediate role in the causal effect chain when assessing the impact of TB on mortality. Standard survival analyses may give biased estimates in the presence of time dependent confounders.[53] All 15 studies controlled in their overall analyses for baseline values of CD4 cell count. This may have resulted in underestimating the effect of TB on mortality by ignoring the effect that TB may have had on mortality through its mediation on CD4 cell counts.[20] Marginal structural models have been introduced to estimate the causal effect of a time-dependent exposure in the presence of time-dependent covariates that may be simultaneously confounders and intermediate variables.[61] In 3 out of 15 studies marginal structural models were constructed[8], [11], [20] and TB status varied in time. The impact of TB on mortality was higher when the marginal structural model had been constructed compared to the HR as obtained by the Cox Proportional Hazard Analyses. These results may provide support to the hypothesis that the pooled effect of TB on mortality in PLWH is an underestimated effect. Other potential time dependent confounders include TB treatment, HAART initiation, viral load or other time varying markers of immunosuppression.

Because of heterogeneity between the studies we have conducted stratified analyses for low CD4 cell count, HAART exposure and region. Meta-regression analyses constitute another possibility to decrease study heterogeneity, but requires information on the main covariates of all included studies, which was unavailable for our meta-analysis.[46] Additionally, we faced the limitation that we only identified 15 eligible studies which decreased the potential for robust conclusions.

Despite the a priori defined in- and exclusion criteria, the main analyses assessing the effect of ‘incident’, ‘prevalent’ or ‘incident + prevalent’ TB on mortality showed substantial heterogeneity across studies, as expressed by I^2^ varying from 45% to 88%. The studies with ‘prevalent TB patients’ [8], [11], [16], [17], [19], [24]–[28], [31] may consist of a heterogeneous cohort of TB patients having already completed several months of TB treatment as well as individuals who are diagnosed at baseline. We used random effect models when heterogeneity was large allowing studies to come from multiple source populations with different distributions between exposure and effect. Because all individual multivariate HRs of the included studies in the main analyses were in similar direction we conclude that the heterogeneity does not impact the direction of the association as found in our meta-analysis, although the heterogeneity may impact on ‘no effect’ versus ‘increased hazard’.

Overall, the quality of the various studies was fairly comparable and excluding the studies with the lowest quality score did not influence the main results. Assessing the quality of studies is sensitive to written information provided in the published manuscripts. Most studies did not get the highest quality score because there was no description on the measurement of TB mortality or a statement on the follow-up time.

### Generalisability

In this meta-analysis we have included studies that selected the cohort based on its participants having a positive HIV status. Although PLWH receiving HAART may be in a more advanced immunosuppressive level than those not receiving HAART, we feel that our choice to only include studies that selected their cohorts based on HIV positive status and not based on the presence of AIDS has enabled us to obtain a population more representative of the average individual infected with HIV.

### Conclusions

This meta-analysis supports evidence that PLWH who develop TB are at increased risk for mortality. Our results are based on observational studies and do not unequivocally establish but imply causality between TB and earlier mortality in PLWH and TB disease.[62] If we admit a causal interpretation then our results would provide an indication of the magnitude of benefit to an individual that could have been expected if the episode of TB had been prevented.[62]

The results emphasize the need for additional studies assessing the effect of preventing TB or early diagnosis and treatment of TB in PLWH on reducing mortality. This may be obtained by providing isoniazid preventive therapy (IPT) which has been associated with decreased mortality in HIV positives co-infected with TB.[63] [64] Furthermore, the risk of TB in PLWH may be reduced by infection control activities to reduce the spread of TB (especially in health facilities) and intensified case finding to pro actively identify TB in people with HIV. [65]

The results of our subgroup analyses in cohorts largely exposed to HAART provide additional support to WHO's revised guidelines which include promoting the initiation of ART for all those with HIV/TB co-infection, irrespective of WHO disease stage or CD4 cell count. [66] We feel that currently available data are insufficient to draw definite conclusions about the causal effect of TB on mortality in PLWH exposed to HAART. This should be further evaluated once the results of additional cohort studies become available in which exposure and adherence to HAART has been documented. Ideally, these studies would prospectively follow individuals starting from HIV seroconversion so that there is information on person years for all phases of HIV infection. TB should be assessed regularly by using standardized approaches including laboratory testing and mortality in PLWH who do and do not develop incident TB should be compared when. [67]

## Supporting Information

Table S1 Study characteristics of studies included in the analysis assessing the effect of tuberculosis on mortality in people living with HIV.
(DOC)

Table S2 Individual results of studies assessing the effect of tuberculosis (TB) on mortality in people living with HIV.
(DOC)
