# Supplementary material for: The Effect of Tuberculosis on Mortality in HIV Positive People: A Meta-Analysis
Source: PLoS One. 2010 Dec 30;5(12):e15241. doi: 10.1371/journal.pone.0015241 (PMC3012688; doi:10.1371/journal.pone.0015241)
Supplement: Table S1 — Study characteristics of studies included in the analysis assessing the effect of tuberculosis on mortality in people living with HIV. (DOC) [file pone.0015241.s001.doc]

Table S1 Study characteristics of studies included in the analysis assessing the effect of tuberculosis on mortality in people living with HIV.

| **First author / prospective (P), retrospective (R), Country** | **Period** | ***N*** | **Population** | **Median age [IQR]**^*^ **/ (SD)† / (range)** | **Median follow-up [IQR] / (range)** | **% of cohort receiving ART‡** | **Median baseline CD4 count [IQR] / (SD) (cells/µL)** | **Median baseline viral load [IQR] Copies/ml** |
| --- | --- | --- | --- | --- | --- | --- | --- | --- |
| Westreich09 [19,32]/ P, South Africa | 2004-2006 | 1,197 TB+ | HIV+ initiating ART | 35 [30-40] | 382 days [134-620] | 100% | 58 [22-116] TB+ | n/a**^¶^** |
|  |  | 6,315 TB- |  | 35 [31-42] | 319 days [110-676] | 100% | 94 [34-165] TB- |  |
| Desai08 [16]/ R, Ethiopia | 2006-2008 | 3,293 | HIV+ | 30 [25-36] | 246 days [7-346] | 8.8% TB+ | 131 [66-254] TB+ | n/a**^¶^** |
|  |  |  |  |  |  | 5.5% TB- | 237 [114-437] TB- |  |
| Fairall08 [8]/ P, South Africa | 2004-2005 | 14,315 | HIV+ ≥ 16 years | 35 [29-41] | 4 months [1-9] | 25.4% | 160 [70-306] | n/a**^¶^** |
| LópezGatell08 [11]/ P, USA | 1984-2005 | 2,882 | HIV1+ males | 35 [31-41] | 5.4 years [2.4-11] | < 24%**^**^** | 533 [365-737] | 12,953 [2453-48,540] |
| Song08 [17]/ R , Cambodia | 2005-2006 | 1,509 | HIV+ | 35 [29-41] | 12 months [365-365 days] | 55% | 52 [20-160] TB+ | n/a**^¶^** |
|  |  |  |  |  |  |  | 162 [58-389] TB- |  |
| LópezGatell07 [20]/ P, USA | 1994-2002 | 1,142 | HIV1+ females | 37 [32-42] | 6 years [3,4-6,8] | 66% | 336 [171-517] | 21,000 [< 4,000-99,500] |
| JonesLopez06 [22]/ P, Uganda | 1993-1995**^††^** | 463 | HIV+ non-anergic ^§§^ | 30 (7) | 884 days (4-1,594) | 0% | 432**^¶¶^** (360) | n/a**^¶^** |
|  |  | 316 | HIV+ anergic ^§§^ | 30 (7) | 575 days (2-1,329) | 0% | 278**^¶¶^** (281) |  |
| Stringer06 [24]/ P, Zambia | 2004-2005 | 12,733 | HIV+ initiating ART | 35 [16-89] | 207 days [103-336] | 100% | 119 [57-193] | n/a |
| Zachariah06 [25]/ P, Malawi | 2003-2005 | 1,584 | HIV+ initiating ART | 35 [30-42] | 298 days [185-473] | 100% | 123 [58-206] | n/a**^¶^** |
| Manas04 [28]/ R, Spain | 1986-1999 | 28 | HIV+/TB+ | 26 (21-31) | 81 months | 0% | 816 (608-1,050) | n/a**^¶^** |
|  |  | 56 | HIV+/TB- | 28 (22-34) | 100 months | 0% | 792 (627-1,030) | n/a**^¶^** |
| VdSande04 [21]/ P, the Gambia | 1992-2001 | 1,134 | HIV1+ > 15 years | 32 [25-40] ^##^ | unknown^***^ | 0%**^†††^** | 195 [70-380] TB+ | n/a**^¶^** |
|  |  | 510 | HIV2+ > 15 years | 39 [30-50] ^##^ | unknown^***^ | 0%**^†††^** | 225 [70-500] TB+ |  |
| Hung03 [26]/ P, Taiwan | 1994-2002 | 125 | HIV+/TB+ initiating ART | 37 (20-75) | 761 days (4-2,2880) | unknown | 37 [0-603] TB+ | 5.58 log_10_ (2.60-5.88) |
|  |  | 591 | HIV+/TB- initiating ART | 34 (15-83) | 616 days (2-2,991) |  | 79 [0-1,202] TB- | 5.14 log_10_ (2.60-5.88) |
| Badri01 [27]/ P, South Africa | 1992-1996**^††^** | 151 | HIV+/TB+ ≥18 | 32 (9.1) | 11.5 months (mean) | 1.9% | ≤ 200: 45% TB+ / 33% TB- | 4.4 log_10_ in sample of 20 TB+ |
|  |  | 451 | HIV+/TB- ≥18 | 32 (8.8) | 14.6 months (mean) | 9.7% | 200-400: 29% TB+ / 34% TB - | 4.4 log_10_ in sample of 20 TB- |
|  |  |  |  |  |  |  | > 400: 26% TB+ / 33% TB- |  |
| Whalen00 [31]/ P, Uganda | 1993-1994**^††^** | 230 | HIV+/TB+ | 29.2 | 19 months | 0% | 376 (311) | n/a**^¶^** |
|  |  | 442 | HIV+/TB- | 30.6 | 19 months | 0% | 431 (367) |  |
| Moreno97 [29]/ R, Spain | 1985-1989**^††^** | 121 | HIV+/PPD+**^#^** | 26 (16-52) | 64 months (1-113) | 56%^§§§^ | 653 (200-2,000) | unknown |

Legend Table S1

* IQR = interquartile range; † SD=standard deviation; ‡ ART = antiretroviral therapy; ¶ n/a = not available: article reports or author has informed that viral load measurements were not available; # PPD+ = purified protein derivative; ** Unknown how many people living with HIV (PLWH) received ART, but 76% of the PLWH entered in the analyses before 1st January 1996 when highly active ART became available, which equals 35% (8295/23,801) person year follow up during highly active ART period.; **^††^** Refers to inclusion period as study period is not specified; ^§§^ Anergic and nonanergic participants in placebo arm TB prophylaxis trial: non-anergic: persons with any induration size (≥ 1mm) to purified protein derivative (PPD) or candidin; anergic: persons with 0mm induration to both PPD and candidin antigens;**^¶¶^** CD4+ cell count is measured in 61% (281/463) nonanergic PLWH and in 53% (169/316) anergic PLWH; ^##^ Age of PLWH and TB disease; ***total personyears of observation is 4,681 years for 1,644 PLWH; **^†††^** at the time of the study ART was not available, if it was known that people received ART through other routes these individuals were excluded from the analyses.; ^§§§^ It is reported that administration of and compliance with ART could not be assessed adequately in all PLWH, but of 72 people, 40 (56%) had been exposed to ART.
