# Supplementary material for: The Effect of Tuberculosis on Mortality in HIV Positive People: A Meta-Analysis
Source: PLoS One. 2010 Dec 30;5(12):e15241. doi: 10.1371/journal.pone.0015241 (PMC3012688; doi:10.1371/journal.pone.0015241)
Supplement: Table S2 — Individual results of studies assessing the effect of tuberculosis (TB) on mortality in people living with HIV. (DOC) [file pone.0015241.s002.doc]

Table S2 Individual results of studies assessing the effect of tuberculosis (TB) on mortality in people living with HIV.

| **First author** | **Outcome measure** | **% TB/ HIV+** | **% mortality TB+/HIV+** | **% mortality**  **TB-/HIV+** | **ID substudy** | **Multivariate HR^*^**  **(95% CI) ^†^** | **Variables adjusted for in**  **multivariate HR^*^** |
| --- | --- | --- | --- | --- | --- | --- | --- |
| Westreich09 [19,32] | Effect of ‘prevalent’ treated TB on mortality in HIV+ initiating highly active antiretroviral therapy | 15.9% (1,197/7,512) | 5.7% (74/1,197) | 3.6% (224/6,315) | Westreich09a | 1.06 (0.75-1.49) ^¶¶^ | list of time fixed and time varying variables**^‡^** |
|  |  | 15.9% (1,197/7,512) | 5.7% (74/1,197) | 3.6% (224/6,315) | Westreich09b | 1.07 (0.81-1.43) | list of time fixed and time varying variables**^‡^** |
| Desai08 [16] | Effect of ‘prevalent’ treated TB on mortality in HIV+ | 18.9% (624/3,293) | 7.7% (42/624) | 4.2% (112/2,669) | Desai08 | 2.77 (1.46-5.26) | age, sex, baseline CD4 cell count, antiretroviral therapy (ART), cotrimoxazole preventive therapy (CPT) |
| Fairall08 [8] | Effect of ‘prevalent/incident’ TB on mortality in HIV+ | 67% (964/14,315) | 24.2% (233/964) | 16.9% (2,258/13,351) | Fairall08a | 1.06 (1.00-1.35) | age, sex, baseline weight, CD 4 cell count, ART, CPT, geographical district |
|  | Effect of ‘incident’ TB on mortality in HIV+ | - | - | - | Fairall08b | 1.13 (0.93-1.36) | age, sex, baseline weight, CD 4 cell count, ART, CPT, geographical district |
|  | Effect of ‘prevalent/incident’ TB on mortality in HIV+ | - | - | - | Fairall08c | 1.12 (0.87-1.44) ^¶¶^ | age, sex, baseline and latest CD4 cell count, ART, CPT, latest weight, |
| LópezGatell08 [11] | Effect of ‘incident’ TB^‡^ on AIDS-related mortality in HIV+ | 0.5% (15/2,882) | 80% (12/15) | 37.9% (1060/2,867) | LópezGatell08a | 2.4 (1.2-4.7) ^¶¶^ | list of time fixed and time varying variables**^§^** |
|  | Effect of ‘incident’ TB^‡^ on AIDS-related mortality in HIV+ | 0.5% (15/2,882) | 80% (12/15) | 37.9% (1060/2,867) | LópezGatell08b | 1.3 (0.6-2.5) | list of time fixed and time varying variables**^¶^** |
| Song08 [17] | Effect of ‘prevalent/incident’ TB on mortality in HIV+ | 27% (401/1,509) | 12.1% (43/355) | 12.3% (103/838) | Song08 | 1.10 (0.73-1.64) | age, sex, baseline CD4 cell count, ART, CPT |
| LópezGatell07 [20] | Effect of ‘incident’ TB on mortality in HIV+ women | 2.1% (29/1,412) | 41.4% (12/29) | 24.8 % (343/1,383) | LópezGatell07a1 (overall) | 2.3 (0.70-7.40) ^¶¶^ | list of time fixed and time varying variables**^#^** |
|  | Effect of ‘incident’ TB on mortality in HIV+ women | - | - | - | LópezGatell07a2 (no HAART) | 2.3 (0.6-9.3) | See list LópezGatell07a1 **^#^**, excluding HAART |
|  | Effect of ‘incident’ TB on mortality in HIV+ women | - | - | - | LópezGatell07a3 (yes HAART) | 2.1 (0.4-12.0) | See list LópezGatell07a1 **^#^**, excluding HAART |
|  | Effect of ‘incident’ TB on mortality in HIV+ women | - | - | - | LópezGatell07b1 (overall) | 1.4 (0.7-2.7) | List of time fixed and time varying variables**^**^** |
|  | Effect of ‘incident’ TB on mortality in HIV+ women | - | - | - | LópezGatell07b2 (no HAART) | 1.9 (0.9-3.8) | See list LópezGatell07b1, excluding HAART |
|  | Effect of ‘incident’ TB on mortality in HIV+ women | - | - | - | LópezGatell07b3 (yes HAART) | 0.6 (0.1-2.7) | See list LópezGatell07b1, excluding HAART |
|  | Effect of ‘incident’ TB on AIDS-related mortality in HIV+ women | - | - | - | LópezGatell07c1 (overall) | 4.0 (1.2-14) ^¶¶^ | List of time fixed and time varying variables**^**^** |
|  | Effect of ‘incident’ TB on AIDS-related mortality in HIV+ women | - | - | - | LópezGatell07c2 (overall) | 2.5 (1.2-5.3) | List of time fixed and time varying variables**^**^** |
| JonesLopez06 [22] | Effect of ‘incident’ TB on mortality in HIV+/PPD- | 9.5% (41/463) | 7.3% (3/41) | 20.9% (88/422) | JonesLopez06a | 6.0 (1.8-20.5) | baseline CD4 cell count, body mass index (BMI), history of HIV-illness, Karnofsky status **^***^** |
|  | Effect of ‘incident’ TB on mortality in HIV+/PPD+ | 7.0% (22/316) | 18.2% (4/22) | 33.3% (98/294) | JonesLopez06b | 6.6 (2.3-19.3) | baseline CD4 cell count, BMI, history of HIV-illness, Karnofsky status**^***^** |
| Stringer06 [24] | Effect of prevalent/incident’ TB on mortality in HIV+ in ART program | 1% (132/12,733) | 9.9% (132/1,1334) | 8.9% (11,399) | - | 1.0 (0.7-1.4) | age, sex, baseline CD4 cell count, BMI, hemoglobin, WHO-stage HIV; non adherence to ART program; time trend |
| Zachariah06 [25] | Effect of ‘prevalent’ TB on mortality in HIV+ on ART | 14.9%(225/1,507) | 10.7% (24/225) | 12.9% (166/1,282) | - | 1.4 (0.92-2.17) | age, sex, CD4 cell count, BMI, WHO-stage |
| Manas04 [28] | Effect of ‘prevalent’ TB on mortality in HIV+^‡‡^ | 100% (28/28) ^‡‡^ | 42.9% (12/28) | 12.5% (7/56) | - | 3.8 (1.5-9.7) | CD4 cell count |
| VdSande04 [21] | Effect of ‘incident’ TB on mortality among HIV1+ | 9.5% (108/1,134) | 76.9% (83/108) | - | VdSande04a (CD4: < 200) | 1.6 (1.2-2.3) | age, baseline CD4 cell count, co-trimaxole use |
|  | Effect of ‘incident’ TB on mortality among HIV1+ | - | - | - | VdSande04b (CD4: 200-500) | 2.5 (1.6-3.8) | age, baseline CD4 cell count, co-trimaxole use |
|  | Effect of ‘incident’ TB on mortality among HIV1+ | - | - | - | VdSande04c (CD4: >500) | 10.0 (5.1-19.7) | age, co-trimaxole use |
|  | Effect of ‘incident’ TB on mortality among HIV2+ | 10% (51/510) | 70.6% (36/51) | - | VdSande04d (CD4: < 200) | 2.9 (1.8-4.7) | age, baseline CD4 cell count, co-trimaxole use |
|  |  |  |  | - | VdSande04e (CD4: 200-500) | 3.0 (1.3-6.8) | age, co-trimaxole use |
| Hung03[26] | Effect of prevalent/incident’ TB on mortality in HIV+ | 17.5% (125/716) | 36% (45/125) | 22.7% (134/591) | Hung03a (overall) | 1.05 (0.73-1.49) | age, gender, baseline CD4 cell count ,year TB diagnosis or enrolled, presence of concurrent AIDS-associated illness at TB diagnosis or enrollment, use of ART, type of ART |
|  | Effect of prevalent/incident’ TB on mortality in HIV+ | - | - | - | Hung03b (before HAART) | 1.18 (0.65-2.14) | See list Hung03a |
|  | Effect of prevalent/incident’ TB on mortality in HIV+ | - | - | - | Hung03c (after HAART) | 0.89 (0.57-1.69) | See list Hung03a |
|  | Effect of prevalent/incident’ TB on mortality in HIV+ | - | - | - | Hung03d (CD4: < 50) | 0.99 (0.65-1.52) | See list Hung03a |
|  | Effect of prevalent/incident’ TB on mortality in HIV+ | - | - | - | Hung03e (CD4:50-<200) | 2.09 (1.06-4.13) | See list Hung03a |
|  | Effect of prevalent/incident’ TB on mortality in HIV+ | - | - | - | Hung03f (CD4: 200-<350) | 1.24 (0.11-14.20) | See list Hung03a |
| Badri01 [27] | Effect of ‘prevalent/incident’ TB on mortality in HIV1+ | 25.9% (158/609) | 31.6% (50/158) | 11.8% (53/451) | - | 2.16 (1.29-3.59) | age, gender, CD4 cell count at TB diagnosis, CPT, ART, risk status, baseline history of AIDS-defining illness |
| Whalen00 [31] | Effect of ‘prevalent’ TB on mortality in HIV+ | 100% (230/230) ^†††^ | 27.4% (63/230) | 19.2% (85-442) | Whalen00a | 1.81 (1.24-2.65) | age, gender, CD4 cell count, tuberculin skin test status, history of HIV-related conditions |
|  | Effect of ‘prevalent’ TB on mortality in HIV+ | - | 46.3%(38/82) | 44.2% (61/138) | Whalen00b (CD4 ≤ 200) | 1.5 (0.99-2.39) | age, gender, history of HIV-related conditions |
|  | Effect of ‘prevalent’ TB on mortality in HIV+ |  | 16.9% (25/148) | 7.9% (24/304) | Whalen00c (CD4 > 200) | 3.0 (1.74-5.66) | age, gender, history of HIV-related conditions |
| Moreno97 [29] | Effect of ‘incident’ TB on mortality in HIV+/PPD+ | 38% (46/121) | - | - | - | 1.88 (1.09-3.27) | CD4 cell count, isoniazid prophylaxis**^***^** |

Legend Table S2

* HR=hazard ratio calculated by Cox Proportional Hazard analyses unless specified otherwise; † CI= confidence interval; ‡ Age, gender, ethnicity, employment, history of ART, history of TB, baseline measures of: pregnancy, peripheral neuropathy, hemoglobin, body mass index, CD4 cell count, WHO stage HIV+, year of enrollment, period treatment initiation; § White ethnicity, age, calendar year, CD4 cell count, and viral load at study entry were included as regressors. Time-varying CD4 cell count, nadir of CD4 cell count, viral load, peak viral load, Pneumocystis jiroveci pneumonia, anti-Pneumocystis jiroveci pneumonia prophylaxis, fever or sweats, *Mycobacterium avium* complex disease, and non-TB AIDS-defining condition were used to compute weights for inverse probability of tuberculosis and censoring; ¶ Fully adjusted for all time fixed and time-varying variables listed before (§ ); # Age, CD4 cell count, HIV-1 RNA level, serum albumin level, and history of non-TB AIDS defining conditions at study entry were included as regressors. Time-varying CD4 cell count, CD4 cell count nadir, HIV type 1 (HIV-1) RNA level, peak HIV-1 RNA level, serum albumin level, HIV-related symptoms (i.e., fever or night sweats, and weight loss), incident non-TB AIDS-defining conditions, anti-Pneumocystis jiroveci pneumonia prophylaxis, previous use of combination and any antiretroviral therapy, HAART use, and low household income were used to compute weights for inverse probability of TB disease, HAART initiation, HAART discontinuation, and censoring. ** Fully adjusted for all time fixed and time-varying variables listed before (#).†† PPD=purified protein derivative test (positive/negative); ‡‡ Retrospective study with TB positive and TB negative cohort; because TB positive cohort does not contribute with non-TB follow up time this is considered as effect of prevalent TB; however despite misdiagnosis we know that non-TB cohort is free of TB; §§ Analyses from the time of TB diagnosis; time to TB infection was included as a discrete time varying covariate, patients on antiretroviral therapy were excluded from the analyses; ¶¶ HR calculated by Marginal Structural Cox Proportional Hazard analyses; **^***^** Multivariate analyses have at least included these variable, but full model has not been reported; ††† Study including TB positive and TB negative cohort.
